# Supplementary material for: Complexome profiling of the Chlamydomonas psb28 mutant reveals TEF5 as an early PSII assembly factor
Source: Plant Cell. 2025 Mar 17;37(6):koaf055. doi: 10.1093/plcell/koaf055 (PMC12164590; doi:10.1093/plcell/koaf055)
Supplement: koaf055_Supplementary_Data [file koaf055_supplementary_data.zip › psb28_interactive.zip!/plots/intensity/qualityPlots/peptides/_Cre08.g375550.t1.2.html]

### Cre08.g375550.t1.2

Trivial name:   
SeqCoverage [median]: 19.90  
Mass: 24.96 kDa  
  
 

| Sequence | MissedCleavage | Mass [Da] | wtIntensity | muIntensity | Score |
| --- | --- | --- | --- | --- | --- |
| NAFLAEALR | 0 | 1003.55 | 576.99 | 2260.96 | 62.00 |
| STAQITVVGEEVKK | 1 | 1487.82 | 1202.95 | 0.00 | 48.11 |
| VIQLQGGTK | 0 | 942.55 | 0.00 | 1299.43 | 75.21 |
| YLGGPSGTISESVFR | 0 | 1568.78 | 748.09 | 1700.87 | 87.18 |
|  |  |  | 2528.0 | 5261.3 |  |
